# Single point mutation in adeno-associated viral vectors -DJ capsid leads to improvement for gene delivery in vivo

**DOI:** 10.1186/s12896-015-0230-0

**Published:** 2016-01-05

**Authors:** Yingying Mao, Xuejun Wang, Renhe Yan, Wei Hu, Andrew Li, Shengqi Wang, Hongwei Li

**Affiliations:** School of Biotechnology, Southern Medical University, 1023 South Shatai Road, Guangzhou, Guangdong 510515 China; Department of Biotechnology, Beijing Institute of Radiation Medicine, Beijing, China; Department of Biomedical Engineering, The Johns Hopkins University School of Medicine, Baltimore, USA

**Keywords:** Point mutation, Adeno-associated virus-DJ, In vivo, Gene delivery, Improvement

## Abstract

**Background:**

Rational design of AAV capsids is a simple method for enhancing AAV transduction efficiency. AAV-DJ is a highly recombinogenic hybrid vector created from DNA shuffling of eight AAV serotypes, which mediates efficient gene expression both in vitro and in vivo. AAV2 and AAV8 are the closest parental vectors of AAV-DJ and it has been reported that mutations on the 137/251/503 ubiquitination or phosphorylation sites of the AAV2 or AAV8 capsid lead to dramatic enhancement of gene delivery. Here, we aimed to find out whether the same point mutations on the AAV-DJ capsid could lead to significant improvement for gene delivery both in vitro and in vivo.

**Results:**

We constructed three single point mutants (K137R/T251A/S503A) of AAV-DJ and the transduction efficiency of these mutants and AAV-DJ were investigated using two reporter gene systems including green fluorescent protein (GFP) and dual-luciferase (Gaussia luciferase and Firefly luciferase). Data indicated that single point mutations T251A/S503A lead to significant improvement of dual-luciferase expression in vivo after tail vein (TV) injection in mice respectively, despite limited enhancement of GFP expression in 293 T, Hela and HepG2 cells in vitro. Moreover, in vivo bioluminescence image and viral genome DNA copy number in tissue analysis showed that these mutants reserved the liver tropism characteristics, consistent with AAV-DJ.

**Conclusion:**

Single point mutations on the 251/503 sites of AAV-DJ capsid can lead to a significant improvement for in vivo gene expression. These enhanced AAV vectors have great potential in gene therapy applications.

## Background

Adeno-associated viruses (AAV) hold great promise in human gene therapy and have been widely used to target liver, muscle, heart, brain, eye, kidney and other tissues in various studies due to its ability to provide long-term gene expression and lack of pathogenicity [1–6]. AAVs belong to the parvovirus family and each contains a single strand DNA flanked by two inverted terminal repeats [7]. There have been reported at least 12 capsid serotypes of AAV vectors (AAV1 to AAV12) and their unique capsid structures enable them to recognize and transduce different cell types and organs. For instance, AAV2-mediated RPE65 gene transfer conferred safe and efficient improvement in retinal function in Leber’s Congenital Amaurosis patients by subretinal injection [8, 9]. AAV8-mediated hFIX gene transfer by a single peripheral-vein infusion consistently leads to long-term expression of the FIX transgene at therapeutic levels without acute or long-lasting toxicity in patients with severe hemophilia B [10].

AAV vector possesses many advantages in gene transfer, but there are still some problems to be solved. AAV infection steps include receptor binding, cell entry, intracellular trafficking, uncoating, second-strand synthesis, vector genome stabilization and so on [11]. The ubiquitin-proteasome degradation machinery has been considered to be a major barrier that negatively affects AAV-mediated gene expression by degrading the viral particles during their intracellular trafficking [12]. Point mutation of surface exposed tyrosine residues of AAV capsid protein was reported as a simple and effective method for evading phosphorylation and subsequent ubiquitination, leading to higher transduction efficiency both in vitro and in vivo [12, 13]. There have also been reported that point mutations on the AAV capsid at specific tyrosine, serine, threonine and lysine residues could lead to significant transduction improvement both in vitro and in vivo [14–18].

AAV-DJ is a variant generated from the libraries of AAV hybrids of eight serotypes by DNA shuffling method [17]. It is able to efficiently transduce a broad range of cell types in vitro but has specific liver tropism in vivo, like AAV8. Moreover, it possesses more ability to evade immune neutralization and thus can efficiently deliver higher quantities of therapeutic DNA [19]. Recently, AAV-DJ has been widely used for gene therapy due to its high efficiency in gene transfer. For example, AAV-DJ vector was used to deliver a knockout construct to fetal pig fibroblasts with a higher targeting frequency than the other naturally occurring serotypes [20]. AAV-DJ mediated homologous recombination (HR) at high efficiency in human keratinocytes and had been used to correct the LAMA3 locus in primary cells, which was not transduced efficiently by the most commonly used AAV serotypes [21].

To further improve the AAV-DJ vector, our study focused on investigating whether point mutations on the capsid of AAV-DJ can increase its transduction efficiency or broaden the tropism both in vitro and in vivo. AAV2 and AAV8 are the closest parental vectors of AAV-DJ and most of the amino acid residues are similar between the capsids of these three vectors. It has been reported that single point mutation on the T251, T503 site of AAV2 or the K137 site of AAV8 capsid leads to dramatic enhancement of gene delivery [14, 15]. In the AAV-DJ capsid, lysine (K) and threonine (T) residues are retained at the 137 and 251 positions as they are in the capsid of AAV8 or AAV2. The single point mutation at the T503 residue, which is equivalent to the T505 residue in the AAV-DJ capsid, has been reported to significantly enhance AAV2-mediated gene delivery [14]. By using the online Phosphorylation and Ubiquitination sites Prediction tools, we found out that the K137 or T251 residue but T505 residue might be an ubiquitination or phosphorylation site, while the residue S503, which is close to the T505 residue and equivalent to the S501 phosphorylation site of AAV2, might be an effective phosphorylation site of AAV-DJ. Thus, we hypothesize that mutation of these predicted sites K137, T251 and S503 in AAV-DJ may cause similar enhancements. In this study, the single point mutants of K137R/T251A/S503A at AAV-DJ capsid were generated respectively and further their differences for in vitro and in vivo transduction efficiency and tropism were evaluated.

## Methods

### Cell cultures

Human embryonic kidney cell line HEK-293 T, human cervical carcinoma cell line HeLa and liver hepatocellular cell line HepG2 were obtained from the American Type Culture Collection (ATCC, Manassas, VA). Cells were maintained as monolayer cultures in Dulbecco’s modified Eagle’s medium (DMEM) (Hyclone, Logan, UT), supplemented with 10 % fetal bovine serum (FBS) (Hyclone), 100 units/ml penicillin, 100 mg/ml streptomycin (Invitrogen, Carlsbad, CA, USA) and maintain in 5 % CO_2_ at 37 °C.

### Construction of AAV-DJ mutants

Phosphorylation and ubiquitination sites on the AAV-DJ capsid were determined using online prediction tools [14, 15]. The predicted sites and conservative amino acid sequences nearby are shown in Tables 1 and 2. Mutations on the AAV-DJ rep/cap plasmid were carried out at K137R, S503A, and T251A by overlapping PCR, and primers are shown in Table 3. A two-stage procedure was performed to achieve the mutations. Briefly, in stage one, two PCR extension reactions were performed in separate tubes for each mutant. One tube contained the forward PCR primer at 5’ site of cap and the reverse primer with point mutation, and the other contained the reverse primer at 3’ site of cap and the forward PCR primer with the point mutation. In stage two, gel-purified PCR products from stage one were used as template at a ratio of 1:1, and a standard PCR was carried out using the forward and reverse primer of cap. The presence of the desired point mutation was verified by DNA sequencing.

**Table 1:** Analysis of the phosphorylation and ubiquitination sites on AAV-DJ capsid

Mutation	Phosphorylation-site prediction	Ubiquitination-site prediction
K137R	―	Yes, high confidence
T251A	Yes	―
S503A	Yes	―

**Table 2:** Conservative amino acid sequences near the three single point mutations on AAV2, AAV8 and AAV-DJ capsids

Serotype	K137R	T251A	S503A
AAV2	EPVKTAP	ALPTYNN	YSWTGAT
AAV8	EGAKTAP	ALPTYNN	FAWTAGT
AAV-DJ	EAAKTAP	ALPTYNN	YSWTGAT

**Table 3:** Primer sequences of the three single point mutations on AAV-DJ capsid

Residue	Sequence (5’ to 3’)	Nucleotide change
K137R	Wild-type primer sequence: TGAGGAAGCGGCTAAGACG	AAG → AGG
	Mutant primer sequence: TGAGGAAGCGGCTAGGACG	
T251A	Wild-type primer sequence: CCCTGCCCACCTACAACAAC	ACC → GCC
	Mutant primer sequence: CCCTGCCCGCCTACAACAAC	
S503A	Wild-type primer sequence: GTGAATACTCGTGGACTGGAG	TCG → GCG
	Mutant primer sequence: GTGAATACGCGTGGACTGGAG	

### Generation of recombinant vectors

Recombinant AAV vectors were generated by polyethyleneimine (PEI)-based triple transfection of 293 T cells. Briefly, twenty 150-mm^2^ dishes 80 % confluent with 293 T cells were transfected with AAV rep/cap, transgene (pAAV-CAG-Gluc-2A-Fluc or pAAV-CBA-GFP), and AAV-helper free (pHelper) plasmids. Cells were collected at 72 h post-transfection, virus was released from the cells by three rounds of freeze–thawing. Crude lysate from all batches was then treated with Benzonase (50U = ml crude lysate) for 1 h at 37 °C. Viral vector stocks were purified by polyethylene glycol (PEG) precipitation followed by double CsCl gradient purification [22]. After three changes of dialysis in virus dialysis buffer (1 × phosphate-buffered saline [PBS], 2 % mannitol, 6 mM MgCl_2_) at room temperature for 6 to 8 h or at 4 °C overnight, vector genome copy titers were determined by quantitative PCR.

### Recombinant AAV vector transduction assays in vitro

To assess the efficacy of the mutant vectors we generated, 293 T, Hela and HepG2 cells were either mock infected or infected with 1000 vector genomes (vg)/cell of AAV-DJ-GFP or its K/S/T mutant vectors. At 48 h post-transduction, GFP expression was observed and imaged on an Olympus Model BX41 fluorescent microscope (Olympus, Tokyo, Japan) and the GFP positive efficiency and mean fluorescent signal intensities (MFI) were measured by flow cytometry (FACS Calibur, BD, USA).

### In vivo gene transfer

Male C57BL/6 mice of 6–8 weeks were obtained and housed in the animal center of the Academy of Military Medicine Science. To evaluate the gene expression level at different time points and the biodistribution of these four vectors after tail vein injection, we used dual luciferase reporter system for the in vivo study. Animals were either mock-injected or injected with 1 × 10^11^ vg each of AAV-CAG-Gluc-2A-Fluc in the same volume via the tail vein (TV). For each group, four mice were used. All animals received humane care and the study protocol complied with the institution’s ethics guidelines.

### Luciferase activity assay

For gene expression level assay, blood was collected via the tail at day 7, 14, 21, 28 and 42 post-vector administrations. The secretory Gaussia princeps luciferase (Gluc) activity was measured using BioLux® Gaussia Luciferase Flex Assay Kit (New England Biolabs) at different time point during the experiment. For each group, the relative light unit (RLU) was determined for all the animals at each time-point. The RLU value is related to Gluc expression.

### In vivo animal bioluminescence image analysis

After the last blood collection, mice were anaesthetized by pentobarbital sodium (1 %, 40 mg/kg mice) via intraperitoneal injection. The D-luciferin substrate (Biotium, Hayward, CA) was injected intraperitoneally at a dose of 150 μg/g. The mice were then placed in a light-tight chamber, and images were generated using a Bioanalytical Instruments (Berthold Technologies, DE). Light was monitored in all of the experiments described at 8–12 min after the substrate injection. The visual output represents the number of photons emitted/second as a false color image where the maximum is red and the minimum is dark blue.

### Genome copy determination

After bioluminescence imaging, mice were killed and tissue samples were collected from each group. Genomic DNA was isolated using the TIANamp Genomic DNA Kit (TIANGEN BIOTECH CO., LTD, Beijing, China) according to the manufacturer’s protocol. Total DNA concentration was determined by spectrophotometry using Nanodrop. Quantitative PCR was used to determine the vector copy numbers in 100 ng of template genomic DNA by amplifying the firefly luciferase sequence (forward primer: ACTGCCTGCGTGAGATTCTC; reverse primer: CAGAGTGCTTTTGGCGAAGA). Glyceraldehyde-3-phosphate dehydrogenase (GAPDH) was used as the housekeeping control gene. Data were captured and analyzed with ABI Prism 7500 sequence detection system version 1.1 software (Life Technologies).

### Statistical analysis

The data presented here were expressed as mean ± standard deviation (SD) and statistical significance was determined by the one-way ANOVA followed by Dunnett post hoc test (compare all columns vs. control group). A *p*-value of < 0.05 was considered statistically significant. *P*-values are indicated by asterisks (***p < 0.001, **p < 0.01, *p < 0.05).

## Results

### Comparison of packaging efficiencies between AAV-DJ and its three single point mutants

To compare the packaging efficiencies between AAV-DJ and its three single point mutants, vectors containing double-strand GFP reporter were packaged by PEI-based triple transfection of 293 T cells. The average titers of the four vectors from at least two packaging experiments were measured by quantitative PCR before vector concentration, which were shown in Table 4. Data indicated that the K137R mutant had 3- to 4-fold higher packaging efficiency while the other two had comparable packaging efficiencies when compared to AAV-DJ, which suggest that the three mutants were compatible modifications on the AAV-DJ capsid.

**Table 4:** The average titers of AAV-DJ and its three single point mutant vectors for packaging double-strand and single-strand reporter genes

Vector	Double-strand GFP reporter	Single-strand luciferase reporter
AAV-DJ	1.08x10^10^vg/ml	6.44x10^10^vg/ml
K137R	4.94x10^10^vg/ml	1.84x10^11^vg/ml
T251A	1.56x10^10^vg/ml	9.95x10^10^vg/ml
S503A	1.44x10^10^vg/ml	8.47x10^10^vg/ml

### AAV-DJ mutants-mediated transduction in vitro

**Fig. 1:**
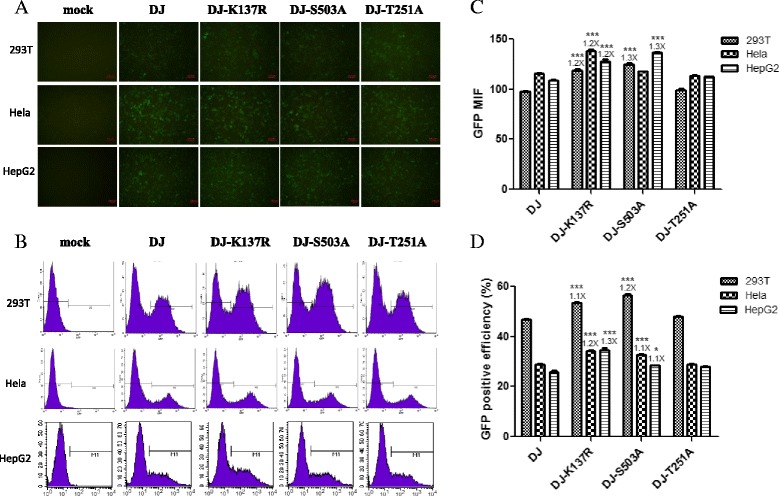
Analysis of AAV-DJ and its mutant vectors transduction efficiency in vitro. 293 T, Hela and HepG2 cells were either mock infected or infected with AAV vectors at an MOI of 1000, and 48 h later GFP expression was observed by fluorescent microscope (**a**) and measured by flow cytometry (**b**). Quantitive analysis of  the mean fluorescent signal intensities (MFI) (**c**) and GFP positive efficiency (**d**) of these cell lines were shown. Levels of significance were determined using one-way analysis of variance. The data are shown as mean values ± SEM

To analyze the transduction potential of the three mutants of AAV-DJ, 293 T, Hela and HepG2 cells were infected with 1000 vector genomes (vg)/cell. At 48 h post-transduction, GFP expression was observed and imaged by fluorescent microscope (Fig. 1a) and the GFP positive efficiencies and mean fluorescent signal intensities (MFI) were measured by flow cytometry. The K137R and S503A point mutations led to 20–30 % enhancement while the T251A mutation led to no significant enhancement of GFP expression when compared to AAV-DJ in these three cell lines (Fig. 1b, c and d). Similar results were obtained when the experiment was carried out using self-complementary GFP expressing vectors (data not shown).

### AAV-DJ mutants led to significant improvement for gene delivery in adult C57BL/6 mice after intravenous injection

**Fig. 2:**
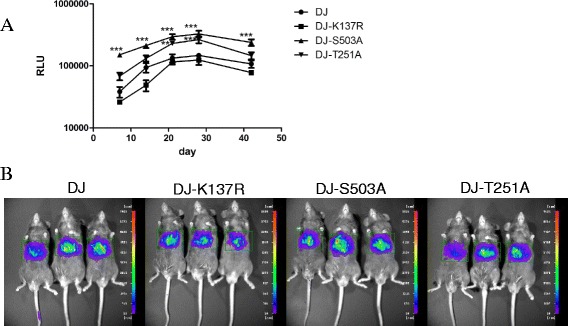
AAV-DJ and its three single point mutant vectors mediated dual-luciferase expression levels in C57BL/6 mice. 1 × 10^11^vg of AAV vectors encoding dual-luciferase were delivered into 6–8 weeks old C57BL/6 male mice via tail vein injection. Gaussia luciferase expression levels were detected at 7, 14, 21, 28 and 42 days after vector injection. The values used are Relative Light Unit (RLU) emitted by the Gaussia luciferase from each animal. Levels of significance were determined using two-way analysis of variance. The data are shown as mean values ± SEM (**a**). For in vivo bioluminescence analysis, images were taken from the ventral aspect after last blood collection and the average expression rang was 100–500 photon/s (ph/s) (**b**)

**Fig. 3:**
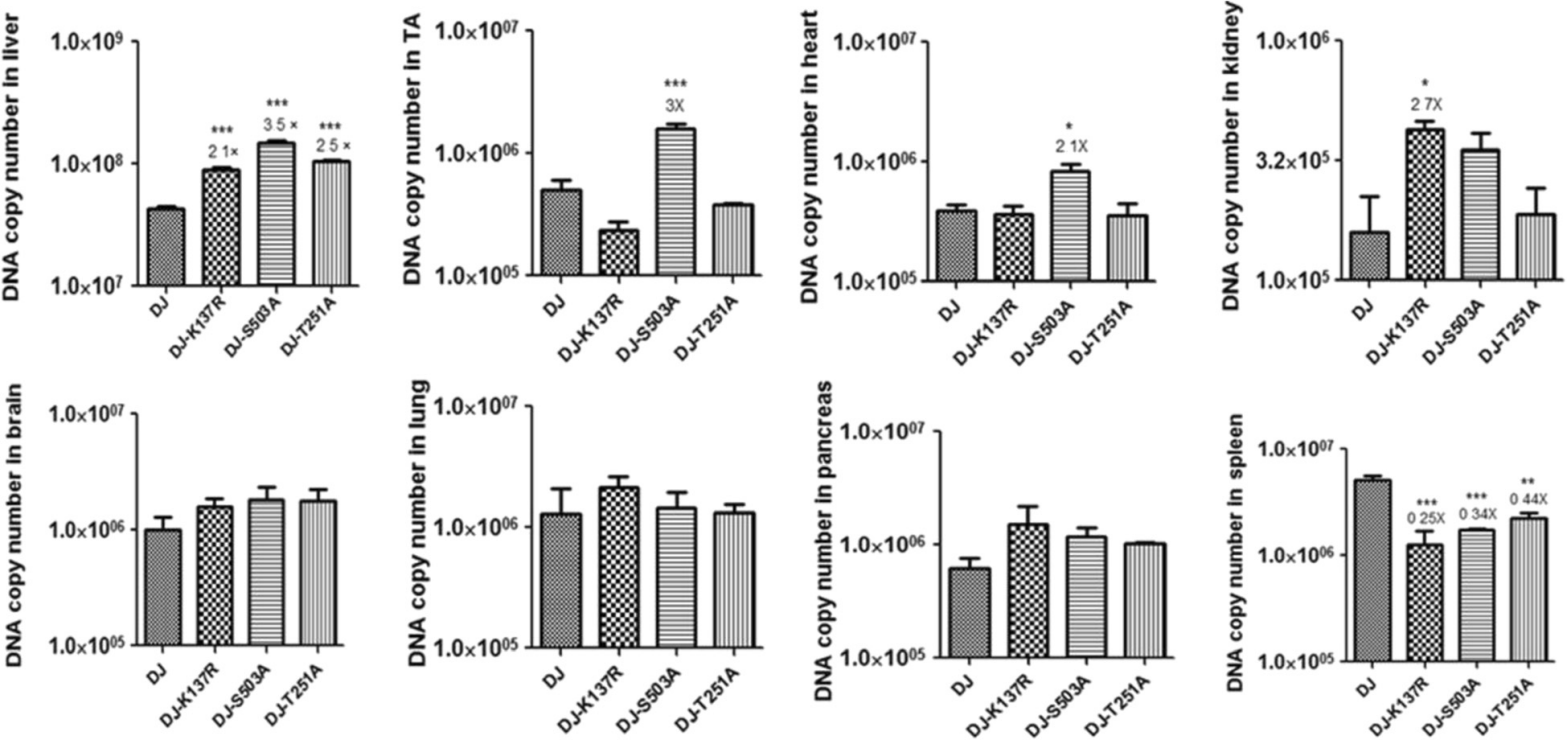
Viral genome DNA copy number analysis in selected tissues. After bioluminescence imaging, animals were killed, genomic DNA was isolated from different tissues, and 100 ng DNA of each tissue sample was used to determine viral genome DNA copy numbers by using primers of Firefly luciferase. Levels of significance were determined using one-way analysis of variance. The data are shown as mean values ± SEM

To study whether the mutants we constructed can lead to differential gene expression in vivo, we used a dual-luciferase reporter system: Gaussia luciferase (Gluc) and Firefly luciferase (Fluc). The Gaussia luciferase is secretory, providing an all-over gene expressing analysis at different time points during the experiment, while the Firefly luciferase is nonsecretory, allowing visualization of localized gene expression via in vivo bioluminescence. In this study, each animal received 1 × 10^11^vg vectors by intravenous injection. First we collected venous blood via the tail at 7, 14, 21, 28 and 42 days after vector injection and evaluated the relative light unit (RLU) emitted by the Gaussia luciferase using a luminometer. As shown in Fig. 2a, the RLU in each animal reached a peak at day 21 and maintained a level of 100,000 ~ 1,000,000 RLU. By two-way ANOVA analysis, the S503A mutant displayed significant enhancement of 2- to 3-fold at each time point (*P* < 0.001), and the T251A mutant showed ~1.8-fold enhancement at day 21 and 28 (*P* < 0.01), while the K137R mutant showed no significant enhancement. To study the in vivo tropism of these vectors, animals were imaged after the last blood collection and the in vivo bioluminescence were analyzed. As shown in Fig. 2b, all vectors exhibited liver tropism. For further evaluation, we compared the viral genome copy numbers in different tissue samples at 42 days after vector injection by using real-time PCR (Fig. 3). The results showed that the K137R, S503A and T251A mutants led to significant enhancement in transduction in liver samples with 2.1-, 3.5-, 2.5-fold higher DNA copy numbers respectively, compared to AAV-DJ. The S503A mutant resulted in 2- to 3- fold higher copy numbers in tibialis anterior (TA) and heart tissues while K137R showed 2.7-fold higher copy numbers in kidney tissue. No significant differences were observed in brain, lung and pancreas. To our surprise, the three mutants showed a significant decrease of 2 ~ 4 fold in DNA copy number than AAV-DJ in spleen tissue.

## Discussion

Many efforts have been made to improve the efficacy of AAV-mediated gene transfer in vitro and in vivo [23–25]. Among these, point mutation in the viral capsid protein seems to be the most simple and universal method. AAV transduction efficiency could be dramatically increased by mutations of particular capsid residues, which allowed the vectors to evade the intracellular phosphorylation and subsequent ubiquitination and proteasome-mediated degradation [26–28]. Point mutation of specific tyrosine, serine, threonine or lysine was reported to significantly increase serotype 2 and 8 AAV-mediated gene transfer both in vitro and in vivo [14, 15, 29]. AAV-DJ, which can efficiently transduce a broad range of cell types in vitro, but has specific liver tropism in vivo, is a chimera of eight AAV serotypes and shares >85 % sequence identity to the parental strains [19]. Thus, we hypothesized that point mutant of specific tyrosine, serine, threonine or lysine in AAV-DJ can further increase its transduction efficiency or change its tropism both in vitro and in vivo. Based on the fact that single point mutation on the T251, T503 residues of AAV2 or the K137 residue of AAV8 capsids can lead to significant gene expression enhancement [14, 15], and through online Phosphorylation and Ubiquitination sites prediction, the K137, T251 and S503 residues of AAV-DJ capsid may be effective ubiquitination or phosphorylation sites, so we decided to generate the following three mutants of AAV-DJ: K137R, T251A and S503A.

As shown in Results, these three mutants of AAV-DJ showed slight enhancement of GFP gene expression in 293 T, Hela and HepG2 cells. There may be several factors that contributed to the in vitro results. As demonstrated by Aslanidi et al., a multiple mutant of AAV2 led to ~24-fold higher efficiency in in vitro gene transduction, which was mainly because the combined mutants had better ability for escaping ubiquitination then resulting in more nuclear translocation and more transgene expression [30]. In our study, we found no significant difference in in vitro gene delivery between AAV-DJ and its mutants, it might because AAV-DJ is a hybrid vector and already has better ability to evade from phosphorylation and ubiquitination [19, 31]. Thus, single point mutations of AAV-DJ may not impact viral entry and cytoplasm/nuclear transgene distribution and is insufficient for enhancing gene delivery in vitro. It remains to be further investigated if multiple mutants of AAV-DJ could lead to enhancement in in vitro gene delivery. Aside from these mutations, AAV-DJ is a hybrid vector with receptor binding regions and major phosphorylation sites on the AAV capsid that may be different from its parental serotypes, thus the same residue mutant may lead to different effects [31]. Moreover, AAV-DJ display superior infectivity in many cell lines than AAV2, which was thought to be the best ex vivo gene delivery AAV vector, so there may be no significant potential for AAV-DJ mediated up-regulation of gene expression in vitro [19, 31]. However, significant enhancements in systemic gene expression level or liver tropism were observed in vivo when the vectors containing the luciferase reporters were injected into mice via tail vein. Two mutants we constructed showed significant enhancement in transduction efficiency in liver, especially for the S503A which exhibited rapid gene expression and maintained higher gene expression level at all the time points investigated. Since the S503 residue of AAV-DJ was equivalent to the S501 residue of AAV2, which turn out to be an effective phosphorylation site of AAV2, it remains to be further studied if the S501A mutant of AAV2 could lead to significant enhancement in AAV2-mediated gene delivery. We would not expect a huge increase for gene expression just as the same mutants of AAV2 did, since AAV-DJ is a hybrid vector and had been strictly selected with human antisera, and has the best ability to avoid phosphorylation in vivo [19, 31].

Recently, some conflicting results were reported on the same residue mutants of different AAV serotypes. For example, the Y to F mutants of AAV2 can dramatically enhance gene transfer and expression both in vitro and in vivo [12, 13, 32]. However, Qiao and co-workers found negative effects when Y to F mutants were applied to AAV8 and AAV9 [33]. Our study on Y to F mutants of both AAV8 and AAV-DJ also displayed minimal enhancement of gene expression in vivo (to be published). Furthermore, Qiao et al. also obtained negative results of K137R on AAV8 [34], which were reported to enhance gene expression by 5 to 10 fold in liver [15]. These inconsistent results may be caused by using different reporters contributing to various measurements of transduction efficiency, incorrect vector titers, dilutions, and/or inconsistent vector potency from batch to batch.

In summary, the AAV-DJ capsid mutants (S503A/T251A) generated in this study led to significant improvements in systemic transgene expression and exhibited liver tropism after tail vein injection in mice. Thus, the enhanced AAV vectors have great potential for future gene therapy applications.

## Conclusions

We successfully constructed three mutants based on phosphorylation or ubiquitination sites on the AAV-DJ capsid. Our results show that the point mutants S503A/T251A can lead to improvement in gene delivery and minimal change in tissue tropism. These enhanced AAV vectors have great potential for future gene therapy applications.
